# NEDD4 expression is associated with breast cancer progression and is predictive of a poor prognosis

**DOI:** 10.1186/s13058-019-1236-7

**Published:** 2019-12-19

**Authors:** Lingfeng Wan, Tao Liu, Zhipeng Hong, You Pan, Steven T. Sizemore, Junran Zhang, Zhefu Ma

**Affiliations:** 1 Department of Breast Surgery, The First Affiliated Hospital, Sun Yat-sen University, No.58 of Zhongshan 2nd Road, Yuexiu District, Guangzhou, 510080 China; 2 Department of Radiation Oncology, The Ohio State University, Arthur G. James Comprehensive Cancer Center and Richard L. Solove Research Institute, 460 West 12th Ave, Columbus, OH 43210 USA; 3 Department of Breast Surgery, Affiliated Quanzhou First Hospital of Fujian Medical University, Quanzhou, 362000 China; 4 Department of Breast Surgery and Plastic Surgery, Cancer Hospital of China Medical University, 44 Xiaoheyan Road, Dadong District, Shenyang, 110042 China

**Keywords:** Breast cancer, NEDD4, Prognosis, Tumor progression

## Abstract

**Background:**

A role for neural precursor cell-expressed developmentally downregulated gene 4 (NEDD4) in tumorigenesis has been suggested. However, information is lacking on its role in breast tumor biology. The purpose of this study was to determine the role of NEDD4 in the promotion of the growth and progression of breast cancer (BC) and to evaluate the clinicopathologic and prognostic significance of NEDD4.

**Methods:**

The impact of NEDD4 expression in BC cell growth was determined by Cell Counting Kit-8 and colony formation assays. Formalin-fixed paraffin-embedded specimens were collected from 133 adjacent normal tissues (ANTs), 445 BC cases composed of pre-invasive ductal carcinoma in situ (DCIS, *n* = 37), invasive ductal carcinomas (IDC, *n* = 408, 226 without and 182 with lymph node metastasis), and 116 invaded lymph nodes. The expression of NEDD4 was analyzed by immunohistochemistry. The association between NEDD4 expression and clinicopathological characteristics was analyzed by chi-square test. Survival was evaluated using the Kaplan–Meier method, and curves were compared using a log-rank test. Univariate and multivariate analyses were performed using the Cox regression method.

**Results:**

NEDD4 promoted BC growth in vitro. In clinical retrospective studies, 16.5% of ANTs (22/133) demonstrated positive NEDD4 staining. Strikingly, the proportion of cases showing NEDD4-positive staining increased to 51.4% (19/37) in DCIS, 58.4% (132/226) in IDC without lymph node metastasis, and 73.1% (133/182) in BC with lymph node metastasis (BCLNM). In addition, NEDD4-positive staining was associated with clinical parameters, including tumor size (*P* = 0.030), nodal status (*P* = 0.001), estrogen receptor status (*P* = 0.035), and progesterone receptor status (*P* = 0.023). Moreover, subset analysis in BCLNM revealed that high NEDD4 expression correlated with an elevated risk of relapse (*P* = 0.0276). Further, NEDD4 expression was an independent prognostic predictor. Lastly, the rates for 10-year overall survival and disease-free survival were significantly lower in patients with positive NEDD4 staining than those in BC patients with negative NEDD4 staining BC (*P* = 0.0024 and *P* = 0.0011, respectively).

**Conclusions:**

NEDD4 expression is elevated in BC and is associated with BC growth. NEDD4 correlated with clinicopathological parameters and predicts a poor prognosis. Thus, NEDD4 is a potential biomarker of poor prognosis and a potential therapeutic target for BC treatment.

## Background

Breast cancer (BC) is the most common cancer in women worldwide, with the incidence increasing in recent years, particularly in developing countries, due to increased life expectancy and the adoption of a western lifestyle [1]. Moreover, BC is the second most common cause of mortality due to cancer, accounting for about 14% of all cancer deaths [2]. Although the combined effects of earlier detection and a range of improvements in treatment have reduced the mortality rate of BC, the incidence for BC is estimated to be increasing globally [3]. Therefore, prevention and therapy of BC remain major public health concerns. The identification of new factors contributing to BC development will be the key to discovering novel targets for BC treatment.

Invasive ductal carcinoma (IDC) is the most common subtype of BC, comprising approximately 60–75% of all breast carcinomas [3]. The current mode for BC development involves a sequential progression from hyperplasia, atypical ductal hyperplasia, ductal carcinoma in situ (DCIS) to ultimately IDC, and eventually metastasis [4, 5]. Various criteria have been used in the clinic to predict the progression of BC. The tumor–node–metastasis (TNM) system is a worldwide classification that describes the stages of BC, often based on tumor size, regional lymph node involvement, and the absence or presence of distant metastasis [6]. TNM stage classifications were developed not only to better understand the clinical behavior of BC, but to also predict the prognosis of similar groups of patients with BC. In addition, several markers have been widely used to predict a BC prognosis in clinical practice, such as the estrogen receptor (ER), progesterone receptor (PR), and human epidermal growth factor receptor 2 (Her2) status, as well as several clinicopathologic features, such as tumor size, histologic grade, and lymph node involvement [7, 8]. Based on the frequently used biomarkers, ER, PR, Her2, and Ki-67, BC is classified into four specific molecular subtypes: luminal A, luminal B, Her2-enriched, and triple negative breast cancer (TNBC) in which ER, PR, and Her2 are all negative [8]. Each subtype of BC responds differently to specific treatments and predicts the prognosis.

E3 ligases are critical components in the ubiquitination cascade, responsible for substrate recognition and modification with specific polyubiquitin chains. The HECT E3 ligase family plays a critical role in regulatory and diverse cellular pathways, operating in and leading to tumor initiation, progression, migration, and resistance to anticancer therapies [9]. NEDD4 E3 protein family members share a similar domain composition: an N-terminal C2 domain, two to four WW domains, and a catalytic HECT domain at the C-terminus [9, 10]. NEDD4 is the product of the neural precursor cell-expressed, developmentally downregulated 4 gene [11]. The cellular function of NEDD4 was initially found to be associated with the regulation of the turnover of the epithelial sodium channel (ENaC) [12]. Subsequently, NEDD4 was demonstrated to be an oncogene due to its role in the negative regulation of the well-known tumor suppressor phosphatase and tensin homolog (PTEN) [13]. Elevated NEDD4 levels and PTEN degradation are observed in various types of human cancer lines [14]. However, no such relationship was observed in BC tissue [15]. In addition, the proto-oncogenic functions of NEDD4 can be attributed to its ability to stabilize the mouse double minute 2 homolog (Mdm2) that exerts its oncogenic activity primarily by suppressing p53 [16]. Moreover, NEDD4 acts as a negative regulator of insulin-like growth factor 1 receptor (IGF-1R) signaling by binding to the adaptor protein, Crb10 [17, 18]. Many other signaling pathways are also regulated by NEDD4, such as the Wnt [19] and notch pathways [20]. In support of its role in promoting oncogenic signaling, NEDD4 overexpression correlates with cell proliferation and transformation [13, 21, 22]. NEDD4 overexpression was detected in malignant gastric, colorectal, and lung cancer cells [21, 23, 24]. In addition, aberrant NEDD4 expression has been implicated in pathogenesis and is associated with an adverse prognosis in gastric cardia adenocarcinoma tumors [25]. Given the role of NEDD4 in cancer growth, progression, and its poor prognosis, NEDD4 as a target is considered to be a promising therapeutic strategy for the treatment of human malignancies [26].

Although NEDD4 expression in BC has been detected in previous studies [15, 27, 28], to our knowledge, the expression of NEDD4 in BC and normal tissue have never been compared. In addition, the role of NEDD4 in BC progression and its prognostic value in BC remains unclear in a clinical context. The goal of this study was to determine the role of NEDD4 in BC growth and progression and to delineate the clinical relevance of NEDD4 to human BC.

Here, we demonstrate that NEDD4 is required for BC growth in vitro. By immunohistochemistry (IHC) analysis, we found that NEDD4 expression was elevated in human BC tissues in comparison to adjacent histologically normal tissues. Most importantly, via the evaluation of NEDD4 expression in a subset of DCIS, IDC, and BC cases with lymph node metastasis (BCLNM) tumors, a gradually increased proportion of NEDD4 staining was observed to the advantage of BC. In support of this observation, the proportion of BC tissue that showed NEDD4-positive staining in TNM stage III was higher than that of TNM stage II, while TNM stage I was lowest. In addition, via a comparison of the expression levels of NEDD4 with known clinicopathologic and molecular features for each patient derived from our database, we found that NEDD4 expression correlated with tumor size, nodal status, and ER and PR status. An analysis of the subset of BC cases with invaded lymph nodes suggested that positive NEDD4 staining is associated with a higher relapse of recurrence compared to a NEDD4-negative staining subset. Lastly, NEDD4 expression is associated with lower overall survival (OS) and disease-free survival (DFS). We concluded that NEDD4 promotes BC growth. NEDD4 expression is associated with BC progression and is a potential biomarker for a poor prognosis. Our study suggests a novel molecular therapeutic target for BC treatment.

## Methods

### Patients and tissue samples

Two cohorts were analyzed in this study: a retrospective cohort of 297 formalin-fixed paraffin-embedded (FFPE) tumor tissues from patients with early stage I–III BC who enrolled in our study from January 1, 2004, to December 31, 2008, including 26 DCIS and 271 IDC cases, as well as 116 FFPE invaded lymph node tissues from patients with BC who had enrolled in this cohort. The secondary cohort consisted of 148 patients with stage I–III BC tumors, which comprised 11 DCIS and 137 IDC cases, as well as 133 paired adjacent normal tissues (ANTs) as negative controls. Of note, 126 cases had BC with lymph node metastasis in the first cohort, and 56 cases made up the second cohort. All samples were taken from the First Affiliated Hospital of Sun Yat-sen University, Guangzhou, China.

ER, PR, Her2, Ki-67, and p53 status were determined from pathology reports of the Pathology Department of the First Affiliated Hospital of Sun Yat-sen University. ER- or PR-positive tissue was defined as more than 10% of tissue staining positive [29]. In the case of Her-2, a fluorescence in situ hybridization assay was performed to evaluate gene amplification in the event of equivocal Her-2 protein expression by IHC. Each patient’s age at diagnosis, menstruation status, tumor grade, tumor size, and nodal status were obtained from medical records. All samples were examined by two independent pathologists. Histological type was based on the TNM system (American Joint Committee on Cancer Classification, 8th, http://www.cancerstaging.org). DFS was defined as the time after surgery to the date of clinical relapse (with histopathology confirmation or radiological evidence of tumor recurrence), a second cancer, or death. Overall survival (OS) was defined as the time from surgery until death from any cause. Uniform guidelines for post-operative follow-up procedures have been described previously [30]. The follow-up deadline was September 28, 2018.

This study was approved by the ethics committee of the First Affiliated Hospital of Sun Yat-sen University. Inclusive criteria are summarized as follows: (1) All patients recruited had unilateral BC and were histologically diagnosed. Adjacent normal breast tissue was selected from an area more than 5 cm from the edge of the tumor. (2) Any patient who had distant metastasis or received preoperative radiotherapy, chemotherapy, hormonal therapy, or any other anticancer therapy before surgery was excluded. (3) Patients with serious complications, such as heart disease, cerebrovascular disease, diabetes, or other malignant diseases, were excluded. (4) Complete clinicopathological data for further analysis were available. (5) All patients were followed up through medical appointments or by telephone.

### Assignment of BC subtype

ER, PR, Her2, and Ki-67 were used to approximate BC subtypes [29]. ER-positive and/or PR-positive and Ki-67 ≤ 20% samples were considered luminal A BC. ER-positive and/or PR-positive and Ki-67 > 20% samples were considered luminal B BC. Her2 positive (independent of ER and PR status) was considered Her2-enriched BC. ER-negative, PR-negative, and Her2-negative samples were considered TNBC. Luminal A and luminal B BC were hormone receptor positive. Her2-enriched and TNBC were hormone receptor negative.

### Tissue microarray construction

The second cohort of tissue samples consisting of 148 cases was prepared for tissue microarray (TMA). All cases were initially selected from paraffin-embedded tumor tissues, and then, sections were reviewed to confirm and select areas for the coring of corresponding blocks. Duplicate tissue cores (1.5-mm diameter) were taken from the central cellular areas of each tumor. The original cohort of cases was arrayed across four blocks. Serial 4-μm sections were cut from TMA blocks.

### IHC

Slides from all patients were stained for NEDD4. Antigen retrieval, blocking procedures, and a modified ImmunoMax method have been previously described [30]. Briefly, slides were deparaffinized and rehydrated, followed by incubation with 3% hydrogen peroxide and methanol to block endogenous peroxidase activity and non-specific protein–protein interactions. Antigen retrieval was performed with citric acid–based buffer at pH 6.0 using a hot plate in a metal container for 15 min before immunostaining. After 1-h blocking for unwanted staining, primary antibody (anti-NEDD4, 1:500, EMD Millipore, Darmstadt, Germany; anti-IGF-1R, 1:50, Abcam, Cambridge, England; anti-PTEN, 1:100, Cell Signaling Technology, Danvers, MA, USA; anti-p-Akt^ser473^, 1:100, Cell Signaling Technology, Danvers, MA, USA) was added at an optimum dilution. A negative control was prepared by the substitution of primary antibody with phosphate-buffered saline (PBS, 5% BSA). All washing steps were performed with PBS alone, along with PBS with 0.1% Tween. To ensure consistent IHC evaluation, slides from a reference tumor previously defined as positive were included in each staining procedure.

### IHC scoring

IHC scoring was performed in a blinded fashion by two independent pathologists. We determined NEDD4 staining in tissues in accordance with an immunoreactive score (IRS) proposed by Remmele and Stegner [31]. IHC scores were determined according to the staining intensity (SI: 0, negative; 1, weak; 2, moderate; 3, strong) and the percentage of positive cells (PP: 0, < 5%; 1, 5–10%; 2, 11–50%; 3, 51–80%; 4, > 81%). An overall immunoreactive score (IRS) was derived by multiplying SI and PP. Slices scoring at least 3 points were classified as showing positive overexpression.

### Cell lines

Human breast cancer cell lines were kindly provided by the Stem Cell Bank, Chinese Academy of Sciences (Shanghai, China). Cell lines were cultured with DMEM (high glucose; Invitrogen, Waltham, MA, USA) supplemented with 10% fetal bovine serum (FBS; Gibco, Waltham, MA, USA) in a humidified atmosphere.

### Small interfering RNA transfections

NEDD4 small interfering (si) RNAs were purchased from RIBOBIO (siRNA#1: TGGCGATTTGTAAACCGAA; siRNA#2: GTGCAAATCATCAGGTTAT; Guangzhou, China). Lipofectamine IMAX (Invitrogen) was used for siRNA transfection. Cells transfected with non-targeting siRNA were used as controls. Transfection efficiencies were validated using quantitative reverse transcription (qRT)–PCR and western blotting.

### Proliferation and colony formation assays

For proliferation, a Cell Counting Kit-8 (CCK8; Dojindo, Kumamoto, Japan) assay was used. Cells were plated into 96-well cell culture clusters at a concentration of 1000 cells/well in a volume of 100 μL after transfections. CCK8 reagents were then added and incubated for 2 h at 37 °C. The absorbance at 450 nm was measured with a microplate reader. For colony formation assays, cells were plated into 6-well cell culture clusters at a concentration of 1000 cells/well in a volume of 2 mL after transfections. After 14 days incubation at 37 °C, cells were fixed with paraformaldehyde for 15 min and stained with 0.5% crystal violet for 30 min. Plates were then washed several times with water, and images of the optical density of colonies were scanned using an optical density scanning analysis system (GS-800; Bio-Rad, Hercules, CA, USA). The number of colonies (> 50 cells) was counted using Image J software.

### Wound healing and transwell assays

For the wound healing assay, cells were plated into a 12-well cell culture cluster at a concentration of 5 × 10^5^ cells/mL. Twenty-four hours later, the cells reached about 90% confluence in a monolayer. A 10-μL pipette tip was then used to scratch a line in the cell monolayer, and the medium was replaced with 1 mL DMEM. The cells were incubated for the indicated times, and images taken under an optical microscope. The gap closure area was measured by Image J software. For transwell assays, cells were plated into an upper transwell chamber at a concentration of 1 × 10^4^ cell/mL in a volume of 200 μL DMEM. The lower chamber of the transwell was filled with 600 μL DMEM plus with 10% FBS. The cells were incubated for the indicated times. After the completion of migration, the cells in the upper chamber were removed with a cotton tip. The cells on the bottom side of the transwell membrane were fixed with 4% paraformaldehyde for 15 min and stained with 0.5% crystal violet for 30 min. Cells were photographed under an optical microscope (DMi8; Leica, Wetzlar, Germany), and the numbers of migrated cells were determined by Image J software.

### Western blot analysis

Total protein was extracted in RIPA lysis buffer. Proteins extracted from cells were resolved using 10% sodium dodecyl sulfate–polyacrylamide (SDS–PAGE) gel electrophoresis, then transferred to a polyvinylidene fluoride membrane (Millipore, Burlington, MA, USA), blocked in 5% non-fat milk (Sigma–Aldrich, St Louis, MI, USA) for 2 h, and blotted with primary antibody (anti-NEDD4, Cell Signaling Technology, Danvers, MA, USA, 1:1000; anti-IGF-1R, Abcam, 1:1000; anti-PTEN, Cell Signaling Technology, 1:500; anti-p-Akt^ser473^, Cell Signaling Technology, 1:2000; anti-GAPDH, Cell Signaling Technology, 1:2000; anti-β-actin, Sigma–Aldrich, 1:5000) overnight at 4 °C. The next day, membranes were incubated with the appropriate HRP-conjugated secondary antibody for 1 h at room temperature. Blots were visualized with an ECL detection kit (Millipore) and analyzed using Image J software.

### Cell line authentication

The authentication of each cell line was confirmed by a 100% match to the reference short tandem repeat profile of the respective cell lines from ATCC.

### Statistical analysis

Statistical analyses were performed using IBM SPSS 23.0 software (Armonk, NY, USA) and GraphPad Prism 7.0 software (San Diego, CA, USA). Data were expressed as mean ± standard deviation (SD) derived from at least three independent experiments. Associations between NEDD4 expression and clinicopathologic data were evaluated using a chi-square test. A comparison of NEDD4 IHC scores between two groups were performed using a Mann–Whitney test. The effects of NEDD4 knockdown or overexpression on cell behavior were examined using a *t* test (two groups) or ANOVA (more than two groups). Survival was calculated using the Kaplan–Meier method, and differences between groups were tested by log-rank test. Univariate and multivariate analyses were undertaken using Cox regression analysis. Correlation analyses were analyzed using the Spearman correlation test. A two-tailed value of *P* < 0.05 was regarded as statistically significant. Data for GSE20685 (microarray-based molecular subtyping of breast cancer) was acquired through Oncomine.com

## Results

### NEDD4 promotes BC growth in vitro

**Fig. 1:**
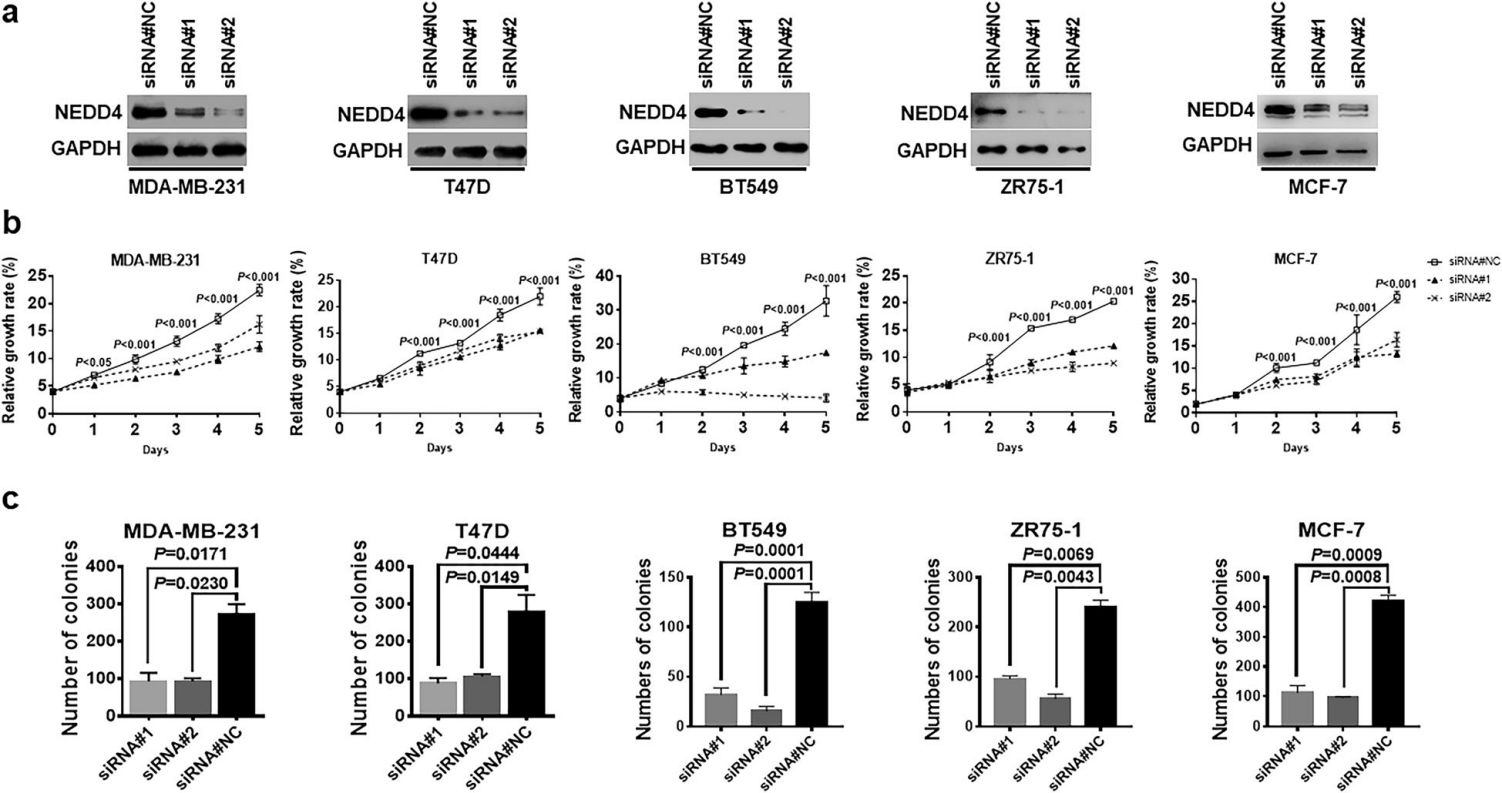
NEDD4 facilitates proliferation in BC cell lines. **a** Knockdown of NEDD4 resulted in reduced NEDD4 protein expression in BC cell lines. **b** CCK8 assays showed that cell proliferation was inhibited after NEDD4 knockdown in BC cell lines (two-way ANOVA). **c** Cell colony formation was significantly retarded after NEDD4 knockdown (one-way ANOVA). NEDD4, neural precursor cell-expressed developmentally downregulated gene 4; BC, breast cancer; CCK8, Cell Counting Kit-8

NEDD4 has a role in promoting the growth of hepatocellular [32] and bladder cancer cell lines [33]. To determine the role of NEDD4 in BC growth, we first examined how NEDD4 expression affects cell growth using BC cell lines from different BC subtypes. NEDD4 was first knocked down by two independent siRNAs in five BC cell lines: luminal A (MCF7, T47D), luminal B (ZR-75-1), and TNBC (MDA-MB-231, BT549; Fig. 1a). Depletion of NEDD4 significantly inhibited cell proliferation in all tested BC cell lines by CCK8 cell proliferation assay (Fig. 1b). This result was further confirmed by colony formation assay. The depletion of NEDD4 in these BC cell lines reduced colony formation compared to control cells with intact NEDD4 expression (Fig. 1c). The impact of NEDD4 on cell growth was verified by MTT cell proliferation assay. A similar result was observed using short hairpin RNAs targeting different coding regions of NEDD4 (Additional file 1: Figure S1). Thus, our results suggest that NEDD4 promotes cell proliferation in BC cell lines.

### NEDD4 is highly expressed in human BC tumors

Given the role of NEDD4 in promoting BC growth, it is expected that NEDD4 is highly expressed in BC. To determine if NEDD4 is highly expressed in human BC tissue, 445 patients diagnosed with early primary BC that were from 2 cohorts of BC were studied. The first cohort was composed of 297 patients with complete clinical and pathologic features and available follow-up data from the period 2004–2008; the median follow-up was 127 months (range 19–171 months). The second cohort was composed of 148 patients with complete clinical and pathologic features as well as 133 paired ANTs from 2014 to 2018. All patients were female. The median age at diagnosis for the 445 patients from both cohorts was 49 years (range from 23 to 80 years). 11.2% of patients were 35 years of age or under at diagnosis, and 40.9% had lymph node metastasis at the time of surgery. In this group, primary therapy included surgical resection in all cases followed by adjuvant hormone, chemotherapy, and radiation therapy in 57.1% (254), 67.6% (301), and 27.6% (123) cases; 13.7% (61) did not receive any form of systemic therapy. The clinicopathologic characteristics of cohorts are provided in Additional file 2: Table S1.

**Fig. 2:**
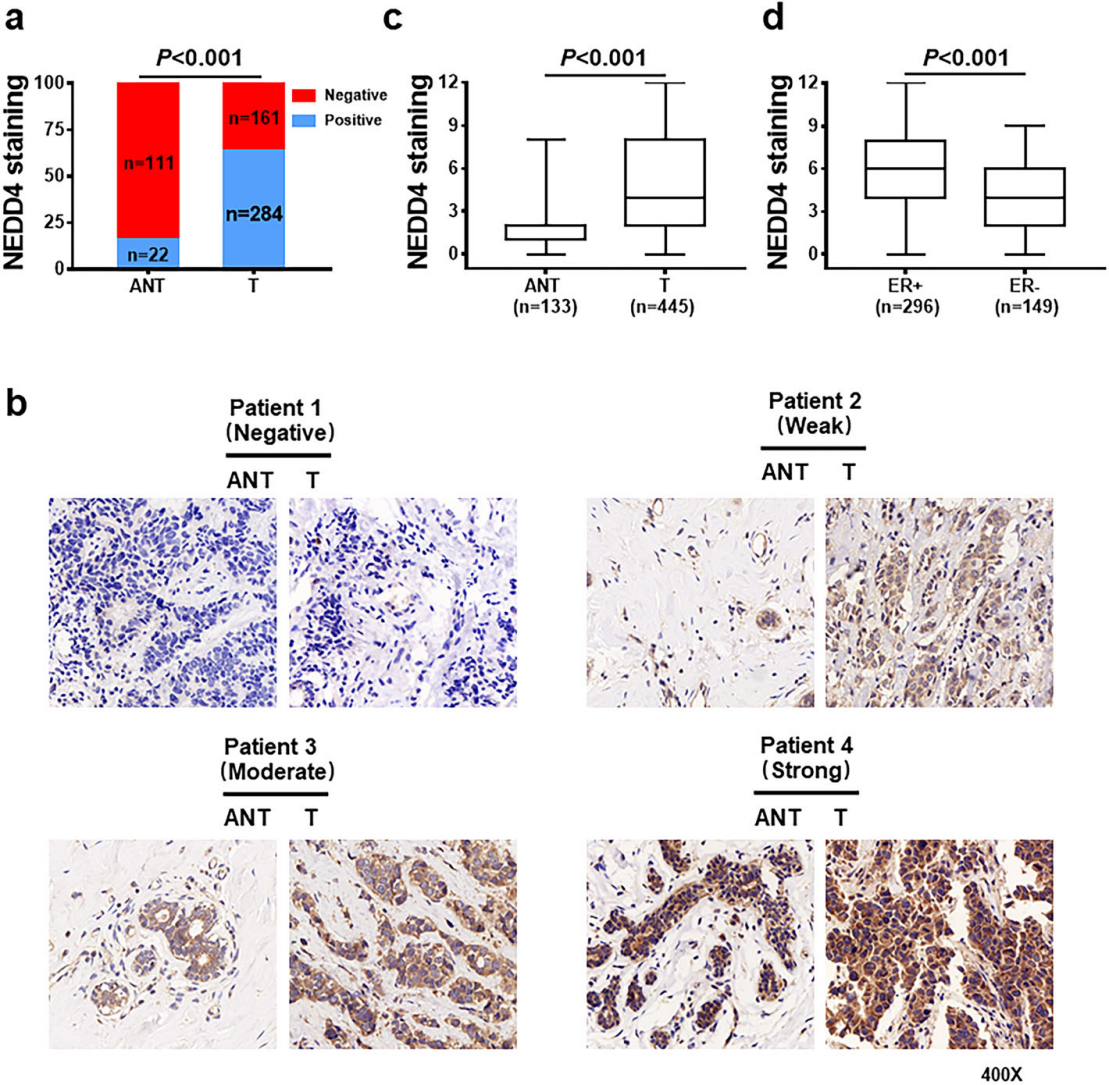
NEDD4 is highly expressed in BC. **a** The percentage of BC tumors with positive NEDD4 staining was compared to that of ANTs (*χ*^2^ test, *χ*^2^ = 94.872, *P* < 0.001). **b** Representative IHC images of different NEDD4 staining grades in BC tumors and paired ANT. **c** The median IRS of BC tumors was significantly higher compared to that of ANTs (Mann–Whitney test, *P* < 0.001). **d** The median IRS was higher in ER-positive BC tumors compared to ER-negative tumors (Mann–Whitney test, *P* < 0.001). NEDD4, neural precursor cell-expressed developmentally downregulated gene 4; ANT, adjacent normal tissue; BC, breast cancer; IRS, immunoreactive score; T, tumor; ER, estrogen receptor; IHC, immunohistochemistry

NEDD4 expression was detected by IHC in all samples prepared for TMAs and FFPE slides using an antibody previously reported to be specific for NEDD4 [13]. Consistent with a previous report [15, 27], NEDD4 staining was found in the cytoplasm of cancer cells. Of all 445 samples, 63.8% of tumor samples were positive for NEDD4 staining, which is similar to a previous report in which NEDD4-positive expression was observed in 55% of BC tumor samples [15] (Fig. 2a). In contrast, only 16.5% of adjacent tissue samples showed positive immunoreactivity for NEDD4 (Fig. 2a). Representative samples of different NEDD4 IHC staining grades in both normal and tumor tissues are shown in Fig. 2b. Thus, the difference in NEDD4 immunoreactivity between tumor samples and adjacent tissues was significant (Fig. 2a, *χ*^2^ = 94.872, *P* < 0.001). The median NEDD4 IRS of BC tumors was significantly higher compared to that of ANT (Fig. 2c, *P* < 0.001). Our study is the first to systemically compare NEDD4 expression in breast cancer and normal tissues. Of all 445 cases, 66.5% (296) were ER positive, including 5.7% (17) DCIS and 94.3% (279) IDC. Noticeably, the median NEDD4 IRS of ER-positive tumors was significantly higher compared to that of ER-negative tumors (Fig. 2d, *P* < 0.001), indicating that NEDD4 expression is associated with ER expression in BC. In support of our result, analysis of the 327 BC patient samples in GSE20685 confirms that NEDD4 mRNA expression is significantly higher in ER-positive vs. ER-negative BC (Additional file 3: Figure S2; *P* < 0.001). Together, the results of Fig. 2 suggest that NEDD4 expression is high in human BC, particularly in ER-positive BC tissue.

Although it is not clear how NEDD4 expression is upregulated in human BC, it has been demonstrated that there is a correlation between NEDD4 protein and mRNA expression in human BC [15]. In support of this, analysis of The Cancer Genome Atlas (TCGA) dataset suggests a strong correlation between NEDD4 protein expression and mRNA in human BC (Additional file 4: Figure S3). Thus, it is most likely that NEDD4 is highly expressed in BC and is related to the transcriptional regulation of NEDD4.

### NEDD4 is associated with clinicopathological features in BC

Next, NEDD4 protein expression was compared with several clinicopathologic variables in BC, such as age, menstruation status, tumor size, histological grade, lymph node involvement, and molecular subtypes. High NEDD4-expressing BC was associated with a large tumor size (*χ*^2^ = 8.973, *P* = 0.030) and a high incidence of lymph node invasion (*χ*^2^ = 10.111, *P* = 0.001; Table 1). In addition, high NEDD4 expression correlated with an ER-positive status (*χ*^2^ = 4.451, *P* = 0.035) and a PR-positive status (*χ*^2^ = 5.197, *P* = 0.023; Table 1). For molecular subtype, 68.8% (44/64) of BC displayed NEDD4-positive staining in luminal A tumors and 66.3% (177/267) in luminal B tumors, both of which were ER/PR positive. In addition, 58.9% (43/73) of Her2-enriched and 48.8% (20/41) of triple negative tumors showed NEDD4-positive staining. However, the difference in NEDD4 expression between hormone receptor positive, Her2-enriched, and triple negative tumors did not reach statistical significance (Table 1, *χ*^2^ = 6.161, *P* = 0.104). In addition, NEDD4 expression showed no obvious relationship with other well-known clinicopathological variables, such as age, menstruation status, tumor grades, Her2 status, or Ki-67 status (*P* > 0.05, respectively; Table 1). Together, the results from Table 1 suggested that NEDD4 expression was associated with the clinicopathological characteristics of tumor size, nodal status, and ER and PR expression.

**Table 1:** Associations between clinicopathological characteristics and NEDD4 expression

Parameter	Status	Total	NEDD4 positive	NEDD4 negative	*χ*^2^	*P*
Age at diagnosis (years)	≤ 35	50	36	14	1.632	0.201
	> 35	395	248	147		
Menopausal status	Premenopausal	240	158	82	0.914	0.339
	Postmenopausal	205	126	79		
Tumor size (cm)	T1a/b	67	36	31	8.973	*0.030*
	T1c	127	73	54		
	T2	220	153	67		
	T3	31	22	9		
Tumor grade	1	39	25	14	2.358	0.308
	2	204	120	84		
	3	144	89	55		
	Unknown	58	50	8		
Nodal status	Positive	182	132	50	10.111	*0.001*
	Negative	263	152	111		
ER status	Positive	296	199	97	4.451	*0.035*
	Negative	149	85	64		
PR status	Positive	306	206	100	5.197	*0.023*
	Negative	139	78	61		
Her2 status	Positive	120	68	52	3.641	0.056
	Negative	325	216	109		
Ki-67 status	≤ 20%	133	82	51	0.385	0.535
	> 20%	312	202	110		
P53 status	Positive	299	190	109	0.291	0.590
	Negative	104	63	41		
	Unknown	42	31	11		
Molecular subtype	Luminal A	64	44	20	6.161	0.104
	Luminal B	267	177	90		
	Her2+	73	43	30		
	TNBC	41	20	21		

Tumor size: 0.1 cm < T1a/b < 1 cm; 1 cm ≤ T1c < 2 cm; 2 cm ≤ T2 < 5 cm; 5 cm ≤ T3

*Abbreviations*: *NEDD4* neural precursor cell-expressed developmentally downregulated gene 4, *ER* estrogen receptor, *Her2* human epidermal growth factor receptor 2, *PR* progesterone receptor, *TNBC* triple negative breast cancer

### NEDD4 is associated with progression of BC

**Fig. 3:**
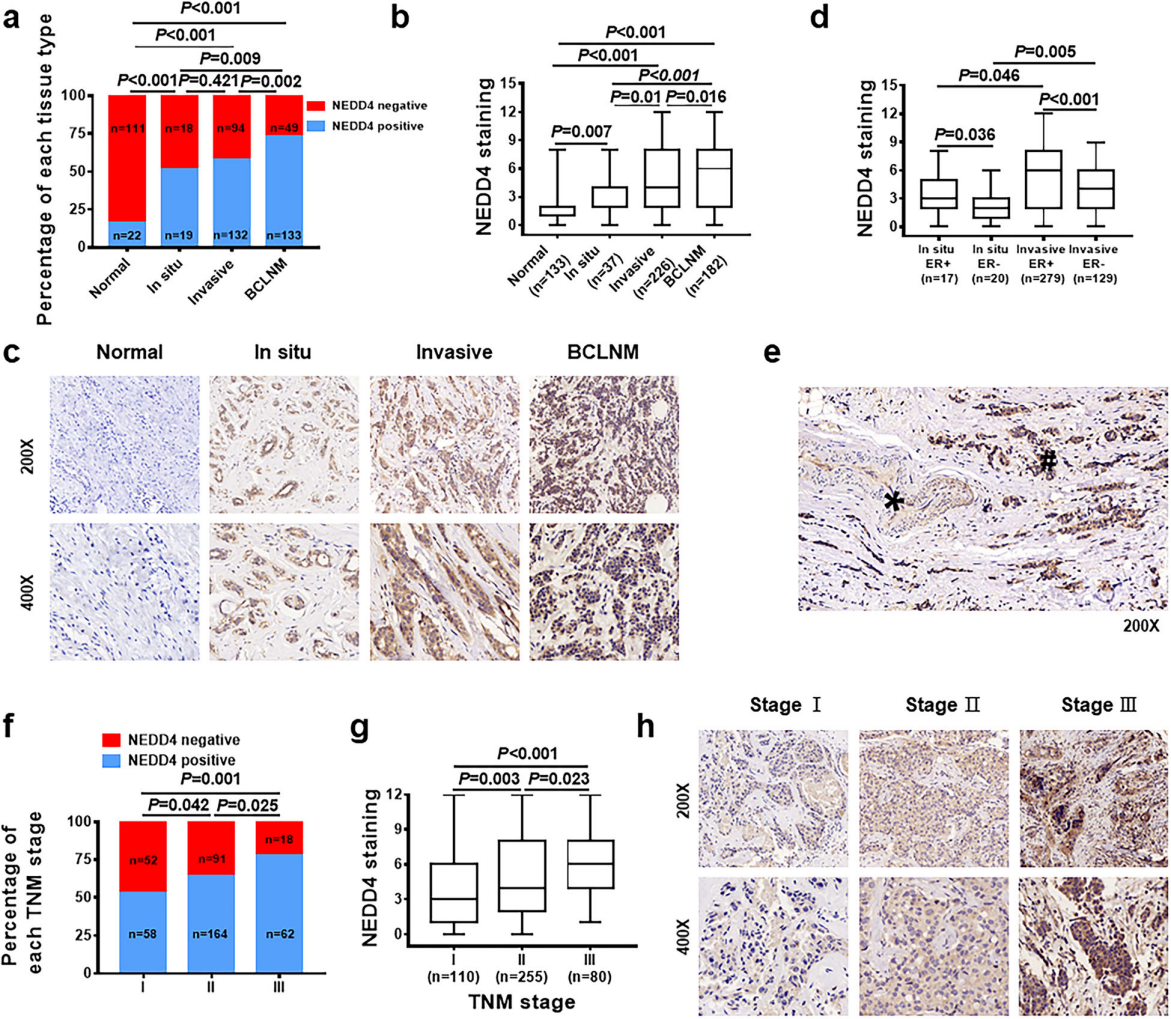
NEDD4 promotes BC progression. **a** The proportion of ANT, DCIS, IDC, and BCLNM tissues showing NEDD4-positive staining is presented. NEDD4-positive expression was more frequent with tumor progression (*χ*^2^ test). **b** The median IRS was significantly increased in ANT, DCIS, IDC, and BCLNM tumors (Mann–Whitney test). **c** Representative IHC images of ANT, DCIS, IDC, and BCLNM are presented. The staining intensity appears higher in invasive lesions. **d** The median IRS is higher in IDC compared with DCIS of the same ER status (Mann–Whitney test). **e** Staining showing that NEDD4 expression is stronger in IDC than that in DCIS in the same tumor sample (*DCIS area, ^#^IDC area). **f** The proportions of TNM stages I, II, and III showing NEDD4-positive staining are presented. NEDD4-positive expression is more frequent with TNM stage (*χ*^2^ test). **g** The median IRS significantly increased with TNM stages I, II, and III (Mann–Whitney test). **h** Representative IHC images of stages I, II, and III are presented. NEDD4, neural precursor cell-expressed developmentally downregulated gene 4; ANT, adjacent normal tissue; DCIS, ductal carcinoma in situ; IDC, invasive ductal carcinoma; BCLNM, breast cancer with lymph node metastasis; IRS, immunoreactive score; IHC, immunohistochemistry; TNM, tumor–node–metastasis; ER, estrogen receptor

In order to determine if NEDD4 expression is associated with BC progression, we subgrouped BC samples based on tumor progression and advantage. We evaluated NEDD4 expression levels in DCIS (*n* = 37), IDC (*n* = 226) without lymph node metastasis, and BC with lymph node metastasis tumors (BCLNM, *n* = 182). NEDD4-positive expression occurred in 51.4% of DCIS, 58.4% of IDC, and 73.1% of BCLNM samples (Fig. 3a). An apparent increase in the rate of NEDD4-positive staining along with progression of the disease occurred, although the difference between DCIS and IDC was not significant (*P* = 0.421). All subtypes of BC in different stages of BC development displayed increased NEDD4 expression compared to ANT (Fig. 3a, DCIS vs. ANT, *χ*^2^ = 19.166, *P* < 0.001; IDC vs. ANT, *χ*^2^ = 62.183, *P* < 0.001; BCLNM vs. ANT, *χ*^2^ = 98.271, *P* < 0.001; IDC vs. DCIS, *χ*^2^ = 0.647, *P* = 0.421; BCLNM vs. DCIS, *χ*^2^ = 6.835, *P* = 0.009; BCLNM vs. IDC, *χ*^2^ = 9.530, *P* = 0.002). Additionally, as the median NEDD4 IRS of ANT, DCIS, IDC, and BCLNM gradually increased, differences between indicated subtypes of BC tissues reached statistical significance (Fig. 3b, DCIS vs. ANT, *P* = 0.007; IDC vs. ANT, *P* < 0.001; BCLNM vs. ANT, *P* < 0.001; IDC vs. DCIS, *P* = 0.01; BCLNM vs. DCIS, *P* < 0.001; BCLNM vs. IDC, *P* = 0.016). Representative IHC images of ANT, DCIS, IDC, and BCLNM are shown in Fig. 3c. In addition, NEDD4 IRS was elevated in the IDC subtype compared with DCIS, regardless of the status of ER (Fig. 3d). However, the rate of NEDD4-positive expression was high in the ER-positive group in both IDC or DCIS subgroups, which further supports the results shown in Fig. 2 and Additional file 3: Figure S2, where NEDD4 expression was associated with ER expression. These results strongly suggest that NEDD4 expression gradually increases during breast tumor progression. A representative sample of NEDD4 staining is displayed in Fig. 3e, in which NEDD4 staining intensity was enhanced in area with IDC compared to an area with DCIS from the same tumor sample (Fig. 3e, *DCIS; ^#^IDC).

In support of the hypothesis that NEDD4 correlated with tumor progression, the NEDD4 expression rate in TNM stage I was 52.7%, which then increased up to 64.3% in stage II and 77.5% in stage III (Fig. 3f, stage II vs. I, *χ*^2^ = 4.402, *P* = 0.042; stage III vs. І, *χ*^2^ = 12.215, *P* < 0.001; stage III vs. II, *χ*^2^ = 4.998, *P* = 0.025). The difference between the median NEDD4 IRS score of each subtype of BC in TNM stages was significant (Fig. 3g, stage II vs. І, *P* = 0.003; stage III vs. І, *P* < 0.001; stage III vs. II, *P* = 0.023). Representative NEDD4 IHC images of each TNM stage in BC are presented in Fig. 3h. Our study is the first to systemically compare NEDD4 expression in BC tissue at different stages of cancer progression. Together, these results suggested that NEDD4 is associated with BC progression and that elevated NEDD4 expression may play an important role in BC development.

### High NEDD4 expression is associated with a poor prognosis in BC

**Fig. 4:**
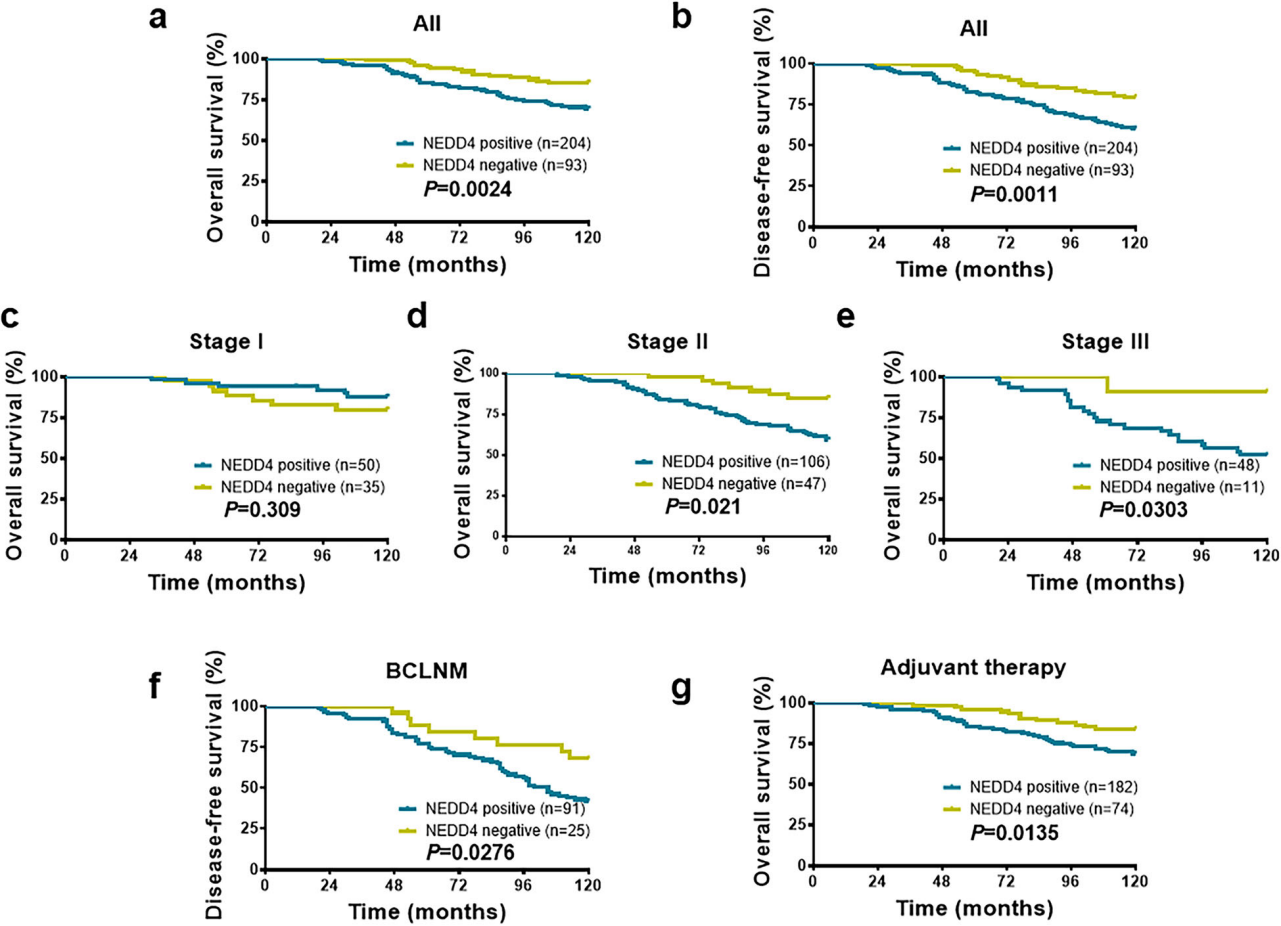
Prognostic impact of NEDD4 in BC. **a**, **b** The Kaplan–Meier analysis indicates that higher NEDD4 expression is correlated with poor OS (log-rank test, *P* = 0.0024) and DFS (log-rank test, *P* = 0.0011). **c**–**e** The OS rate of patients with BC in different TNM stages. **f** High NEDD4 expression is correlated with a high risk of relapse in invaded lymph node tumors (log-rank test, *P* = 0.0276). **g** The OS rate of patients with BC who received adjuvant therapies. NEDD4, neural precursor cell-expressed developmentally downregulated gene 4; BCLNM, breast cancer with lymph node metastasis; DFS, disease-free survival; OS, overall survival; TNM, tumor–node–metastasis; BC, breast cancer

We next determined the prognostic value of NEDD4 expression. The association of NEDD4 expression with overall survival (OS) and DFS of patients with BC was evaluated by the Kaplan–Meier analysis. This analysis was based on a 10-year follow-up of 297 patients since those patients had complete follow-up data. As shown in Fig. 4, patients with positive NEDD4-expressing BC had a shorter OS (Fig. 4a, *P* = 0.0024) and DFS (Fig. 4b, *P* = 0.0011) than patients with negative NEDD4-expressing BC.

The association of NEDD4 expression with OS in each TNM stage BC was further analyzed with the Kaplan–Meier analysis. As shown in Fig. 4c–e, NEDD4 expression was inversely associated with OS in TNM stages II and III patients (Fig. 4d, e, *P* = 0.021, *P* = 0.0303, respectively), but not stage І patients (Fig. 4c, *P* = 0.309). These results clearly show that patients with NEDD4-positive staining BC in stages II and III had a lower survival rate during follow-up, suggesting that NEDD4 expression may be a feasible index for predicting a poor survival rate in patients with BC. Additionally, in the subgroup of BC patients with invaded lymph nodes, those with NEDD4-positive staining showed a high relapse during the follow-up period of 10 years compared with the NEDD4-negative expression group (Fig. 4f, *P* = 0.0276). These results suggested that NEDD4 expression is associated with a poor prognosis and is linked to a high risk of relapse in BC.

Next, we investigated the relationship between NEDD4 expression and rates of OS in the following subsets of patients with BC: those who received or did not receive adjuvant therapy, ER positive or negative, and Her2 positive or negative. NEDD4 expression was associated with a low OS rate in patients who received adjuvant therapy (Fig. 4g, *P* = 0.0135). Interestingly, a statistical correlation between NEDD4 expression and OS was not found in ER-positive breast tumors (Additional file 5: Figure S4a, *P* = 0.0865), while high NEDD4 expression was associated with a lower OS rate in ER-negative patients (Additional file 5: Figure S4b, *P* = 0.0204). Additionally, no association between NEDD4 expression and OS was observed in Her2-positive BC patients (Additional file 5: Figure S4c, *P* = 0.0702), which is consistent with a previous publication showing that NEDD4 expression was not associated with clinical outcomes in Her2-positive BC patients [27]. However, an association was found between NEDD4 expression and OS in patients with Her2-negative BC (Additional file 5: Figure S4d, *P* = 0.0355).

In support of our results, analyses of the 327 BC patient samples in GSE20685 revealed that NEDD4 mRNA expression is highly prognostic of OS (*P* = 0.04835) and distant metastasis-free survival (*P* = 0.0033) in the ER-negative patient population (Additional file 6: Figure S5c,d). Intriguingly, NEDD4 expression is not prognostic of survival in the ER-positive patient population of this cohort, nor is NEDD4 prognostic when survival is examined in combined ER-negative and ER-positive populations of the same cohort (Additional file 6: Figure S5a,b,e,f). These results suggest that the prognostic utility of NEDD4 may be strongest in ER-negative BC.

Lastly, we used a Cox proportional hazard model to determine the prognostic value of NEDD4. NEDD4 immunoreactivity, patient’s age, tumor size, histological grade, and nodal status were chosen as risk variables since all are potential factors affecting a poor prognosis for BC. Hazard ratios are indicated in Table 2. In univariate and multivariate analyses, NEDD4 expression, tumor grade, and nodal status were three independent factors related to the OS rate of BC (Table 2 (a); HR = 2.353, 95% CI = 1.550 to 3.572, *P* < 0.001; HR = 2.003, 95% CI = 1.333 to 3.007, *P* = 0.001; HR = 2.105, 95% CI = 2.212 to 4.360, *P* < 0.001, univariate analyses, respectively; HR = 2.134, 95% CI = 1.394 to 3.268, *P* < 0.001; HR = 2.186, 95% CI = 1.443 to 3.310, *P* < 0.001; HR = 2.678, 95% CI = 1.818 to 3.970, *P* < 0.001, multivariate analyses, respectively). With regard to DFS, using a Cox regression model, we found that NEDD4 expression, lymph nodal status, and tumor grade were three independent factors related to DFS (Table 2 (b); HR = 2.407, 95% CI = 1.667 to 3.475, *P* < 0.001; HR = 3.762, 95% CI = 2.771 to 5.108, *P* < 0.001; HR = 1.752, 95% CI = 1.222 to 2.510, *P* = 0.002, univariate analyses, respectively; HR = 2.185, 95% CI = 1.507 to 3.162, *P* < 0.001; HR = 3.289, 95% CI = 2.310 to 4.683, *P* < 0.001; HR = 1.796, 95% CI = 1.246 to 2.589, *P* = 0.002, multivariate analyses, respectively). Last, univariate and multivariate Cox regression analyses suggested that NEDD4 expression and tumor grades were independent risk factors for relapse in BC patients with invaded lymph nodes (Table 2 (c); HR = 2.512, 95% CI = 1.460 to 4.321, *P* = 0.001; HR = 1.814, 95% CI = 1.162 to 2.932, *P* = 0.009; HR = 1.798, 95% CI = 1.162 to 2.780, *P* = 0.008, univariate analyses, respectively; HR = 2.834, 95% CI = 1.636 to 4.911, *P* < 0.001; HR = 2.033, 95% CI = 1.293 to 3.197, *P* = 0.002; HR = 1.861, 95% CI = 1.143 to 3.029, *P* = 0.013, multivariate analyses, respectively). Together, the data presented in Fig. 4 and Table 2 suggest that NEDD4 expression is inversely associated with a poor prognosis in BC. NEDD4 expression is an independent predictive factor for OS and DFS, particularly in BC patients with invaded lymph node metastasis.

**Table 2:** Univariate and multivariate analyses

a. Univariate and multivariate analyses of associations between clinical parameters, NEDD4 status, and overall survival					
Overall survival parameter	Comparison	Univariate		Multivariate	
		HR (95% CI)	*P*	HR (95% CI)	*P*
Age at diagnosis (years)	> 35 vs. ≤ 35	0.648 (0.373–1.123)	0.122	0.887 (0.506–1.556)	0.676
Tumor size (cm)	T2 vs. T1	1.087 (0.554–2.135)	0.808	2.166 (1.067–4.397)	0.032
	T3 vs. T1	1.333 (0.692–2.567)	0.391	1.625 (0.840–3.143)	0.149
Tumor grade	2 vs. 1	2.003 (1.333–3.007)	*0.001*	2.186 (1.443–3.310)	*0.000*
	3 vs. 1	0.728 (0.493–1.074)	0.109	1.015 (0.664–1.554)	0.944
NEDD4 expression	Post vs. neg	2.353 (1.550–3.572)	*0.000*	2.134 (1.394–3.268)	*0.000*
Nodal status	Post vs. neg	2.105 (2.212–4.360)	*0.000*	2.678 (1.818–3.970)	*0.000*
b. Univariate and multivariate analyses of associations between clinical parameters, NEDD4 status, and disease-free survival					
Disease-free survival parameter	Comparison	Univariate		Multivariate	
		HR (95% CI)	*P*	HR (95% CI)	*P*
Age at diagnosis (years)	> 35 vs. ≤ 35	0.671 (0.408–1.105)	0.117	0.957 (0.577–3.167)	0.865
Tumor size (cm)	T2 vs. T1	0.784 (0.466–1.379)	0.399	1.501 (0.831–2.713)	0.178
	T3 vs. T1	1.205 (0.703–2.065)	0.497	1.482 (0.0.863–2.546)	0.154
Tumor grade	2 vs. 1	1.752 (1.222–2.510)	*0.002*	1.796 (1.246–2.589)	*0.002*
	3 vs. 1	0.635 (0.453–0.89)	0.008	1.057 (0.723–1.543)	0.776
NEDD4 expression	Post vs. neg	2.407 (1.667–3.475)	*0.000*	2.185 (1.507–3.167)	*0.000*
Nodal status	Post vs. neg	3.762 (2.771–5.108)	*0.000*	3.289 (2.310–4.683)	*0.000*
c. Univariate and multivariate analyses of associations between clinical parameters and NEDD4 status and disease-free survival of BCLNM					
Disease-free survival parameter	Comparison	Univariate		Multivariate	
		HR (95% CI)	*P*	HR (95% CI)	*P*
Age at diagnosis (years)	> 35 vs. ≤ 35	1.334 (0.697–2.550)	0.384	1.328 (0.688–2.561)	0.397
Tumor size (cm)	T2 vs. T1	1.455 (0.753–2.811)	0.265	1.142 (0.553–2.356)	0.720
	T3 vs. T1	1.127 (0.612–2.077)	0.701	1.074 (0.529–1.991)	0.821
Tumor grade	2 vs. 1	1.814 (1.162–2.932)	*0.009*	2.033 (1.293–3.197)	*0.002*
	3 vs. 1	1.798 (1.162–2.780)	*0.008*	1.861 (1.143–3.029)	*0.013*
NEDD4	Post vs. neg	2.512 (1.460–4.321)	*0.001*	2.834 (1.636–4.911)	*0.000*

*Abbreviations*: *NEDD4* neural precursor cell-expressed developmentally downregulated gene 4, *BCLNM* breast cancer with lymph node metastasis, *CI* confidence interval, *HR* hazard ratio, *Neg* negative, *post* positive

Given that the rate of NEDD4-positive staining is the highest in the BCLNM subgroup and that NEDD4 expression is associated with OS and DFS, particularly in this subgroup, this suggests that NEDD4 may promote metastasis. To determine the role of NEDD4 in cancer cell migration in vitro, we conducted wound healing and transwell assays. We found that similar wound healing recovery was noted in cells, with or without knockdown of NEDD4, using two different cell lines, MDA-MB-231 and T47D. NEDD4 knockdown (Additional file 7: Figure S6a,b) had no obvious effect on the wound healing process compared to control cells with intact NEDD4. In addition, we also measured migration by transwell assay. Consistent with wound healing assay results, NEDD4 knockdown had no effect on cell migration in the transwell assay (Additional file 7: Figure S6c). Thus, NEDD4 is not essential for cell migration in vitro. As tumor metastasis is a complicated multistep process, it may be that NEDD4 influences the metastatic process via some mechanism other than cell migration.

### NEDD4 expression is associated with IGF-1R/Akt pathway

**Fig. 5:**
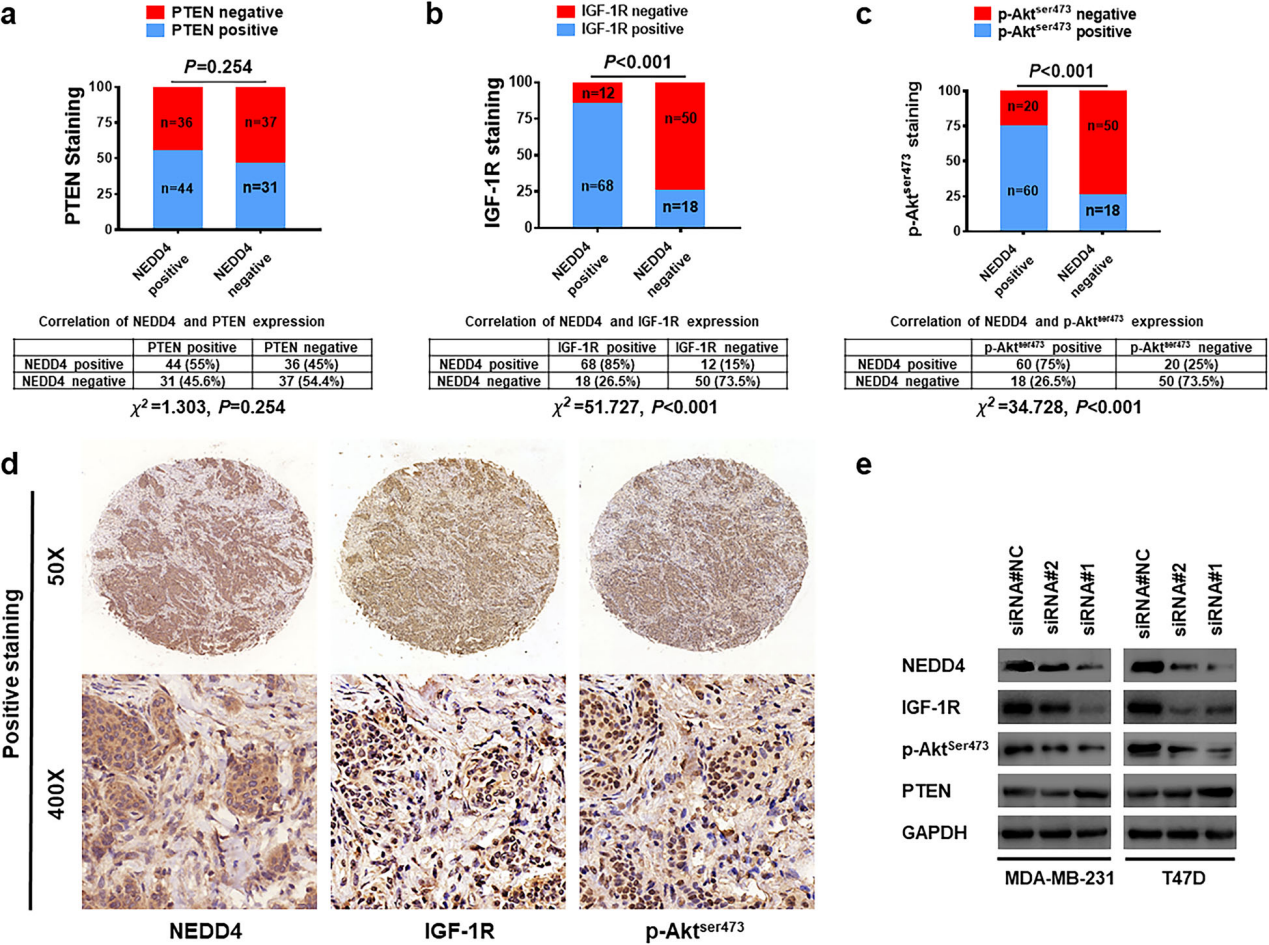
NEDD4 expression is associated with IGF-1R/Akt pathway **a** The expression of NEDD4 and PTEN in BC tissue. A TMA containing 148 BC tissue samples was immunohistochemically stained with anti-NEDD4 and anti-PTEN. **b** The expression of NEDD4 and IGF-1R in BC tissue. A TMA containing 148 BC samples was immunohistochemically stained with anti-NEDD4 and anti-IGF-1R. **c** The expression of NEDD4 and p-Akt^ser473^ in BC tissue. A TMA containing 148 BC samples was immunohistochemically stained with anti-NEDD4 and anti-p-Akt^ser473^. **d** Representative immunostaining patterns for serial sections of the same tumor for NEDD4, IGF-1R, and p-Akt^ser473^. **e** Protein expression level of indicated proteins was detected by western blots in BC cells, with or without NEDD4 knockdown. GAPDH was used as a control. NEDD4, neural precursor cell-expressed developmentally downregulated gene 4; BC, breast cancer; PTEN, phosphatase and tensin homolog; TMA, tissue microarray; IGF-1R, insulin-like growth factor receptor

To determine the potential molecular mechanisms by which NEDD4 promotes breast tumor growth and progression, we determined the expression of NEDD4 along with PTEN, IGF-1R, and p-Akt by IHC using a TMA that consisted of 148 samples of early-stage primary invasive breast cancer. Positive PTEN staining was found in 55% (44/80) of BC samples with positive NEDD4 staining, which is comparable to the rate of 45.6% (31/68) observed in BC tissue samples with negative NEDD4 staining (Fig. 5a). This suggests a lack of correlation between NEDD4 and PTEN expression in human BC tissue. As for IGF-1R expression, 85% (68/80) of NEDD4-positive staining breast carcinomas were found to stain positive for IGF-1R whereas only 26.5% (18/68) of BC tissue with negative NEDD4 staining stained positive for IGF-1R (Fig. 5b). A similar pattern was also observed for p-Akt^Ser473^ staining. Seventy-five percent (60/80) of NEDD4-positive BC samples stained positively for p-Akt^Ser473^ whereas it was only 26.5% (18/68) in NEDD4-negative BC samples (Fig. 5c). IHC staining revealed that IGF-1R and p-Akt^Ser473^ levels were found to be consistently higher in areas of high NEDD4 protein levels (Fig. 5d). In support of the results obtained from IHC staining, knocked down NEDD4 led to decreased IGF-1R and p-Akt^Ser473^, even in the T47D cells that harbor an activating PI3K mutation that is constitutively active [34] (Fig. 5e). As for PTEN protein, only dramatic NEDD4 knockdown by siRNA #1 led to increased PTEN; however, no significant alteration in PTEN protein expression was observed in cells with moderate NEDD4 knockdown by siRNA#2 (Fig. 5e). Given that NEDD4 knockdown by both siRNAs resulted in the slow growth of BC (Fig. 1) and that there is no correlation between NEDD4 and PTEN protein expression in BC tissue samples (Fig. 5a), the results displayed in Fig. 5 suggest that NEDD4 promotes BC progression via the activation of IGF-1R/Akt signaling, perhaps independently of PTEN.

## Discussion

### NEDD4 expression is associated with BC growth and progression

Our study is the first to systemically assess the association of NEDD4 with BC cell growth, and progression/prognosis in early-stage BC. We found that NEDD4 is required for the proliferation of BC (Fig. 1), which supports the observation that NEDD4 expression was elevated in human BC tumor tissue compared to normal BC tissue (Fig. 2). In addition, NEDD4 expression was associated with tumor size, TNM stage, nodal status, and ER and PR status (Table 1). However, NEDD4 expression is not associated with a Her2, Nottingham Histologic Grade, and Ki-67 [15]. Of note, although NEDD4 expression correlated with ER status (Fig. 2d, Fig. 3d, Additional file 3: Figure S2), NEDD4 seems to have no influence on OS in ER-positive patients (Fig. 3a, Additional file 5: Figure S4a, Additional file 6: Figure S5e). This may be explained by the fact that in general, most patients with ER-positive tumors were sensitive to hormone therapy and CDK4/6 inhibitors [35]; ER-positive patients usually showed satisfactory outcomes after hormone therapy [36, 37], which overshadowed the effect of NEDD4 overexpression. Our study is consistent with a previous publication showing that NEDD4 expression is associated with the status of ER expression [15]. However, the nature of the association between ER and NEDD4 expression remains unknown and needs to be determined in future.

Cumulative evidence suggests that NEDD4 is linked to tumor progression in several human cancers, such as gastric carcinoma, hepatoma carcinoma, bladder cancer, and prostate cancer [25, 32, 33, 38]. Based on our studies, NEDD4 expression gradually increases in line with BC progression, from normal tissues, DCIS, IDC without lymph node metastasis to BCLNM tumors, which is a well-established clinical model for BC progression [5]. In addition, gradually increased NEDD4 expression was observed in stages I, II, and III BC samples. Thus, our studies strongly suggest that NEDD4 expression is associated with BC progression. Indeed, this hypothesis is further supported by previous studies that showed high NEDD4 expression promoted tumor progression in lung cancer [39] and gastric cardia adenocarcinoma [25].

### High NEDD4 expression is a biomarker for poor outcomes

Previous work has demonstrated that NEDD4 is associated with poor survival in gastric carcinoma [25] and hepatocellular cancer [40]. In our study, we found that high NEDD4 expression was associated with a poor post-surgery prognosis in patients with BC, as reflected by their OS and DFS. Therefore, NEDD4 expression may serve as a predictive biomarker of a poor prognosis for BC. Interestingly, no correlation between NEDD4 expression and OS was found in the datasets from TCGA (as of September 23, 2019, data not shown). Several reasons may exist for this discrepancy, including the heterogenous expression of NEDD4 in tumors, intratumoral heterogeneity, and significant variability in the NEDD4 antibodies and scoring systems used. To reach a firm conclusion may require additional investigations in future.

We also found that NEDD4 expression is an independent factor for a poor prognosis along with two well-known predictive factors, tumor grade, and nodal status (Table 2 (a, b)) but independent from established prognostic factors such as tumor size, margin status, and menstruation status [41]. Further analysis in a subset of BCLNM suggested that patients with NEDD4-positive staining correlated with a high risk of relapse (Fig. 4f). In addition, the highest proportion of NEDD4-positive staining was found in BCLNM tissues (Fig. 3a). An analysis of GSE20685 indicated that high NEDD4 expression is associated with lower distant metastasis-free survival in women with ER-negative BC (Additional file 6: Figure S5d). Such results point to the role of NEDD4 in BC metastases. However, no obvious role for NEDD4 in cell migration was observed in in vitro assays suggesting that NEDD4 exerts its influence on BC metastasis through some other mechanism.

In general, ER-negative tumors are more aggressive and metastatic [42] compared to ER-positive tumors. NEDD4 expression is associated with lower OS in a subset of ER-negative patients. An identical trend was also observed in a Her2-negative subgroup, whereas NEDD4 expression was not associated with OS in a subset of BC that was Her2 positive. This is in line with a previous study showing that NEDD4 expression did not predict the efficiency of adjuvant trastuzumab therapy in Her2-positive BC patients [27]. The current treatments for ER-negative and/or Her2-negative BC tumors mainly rely on traditional cytotoxic therapies, which directly or indirectly cause cell DNA damage. Therefore, NEDD4 expression may be a predictor of a poor prognosis in the subset of patients with ER-negative/Her2-negative BC.

### The mechanisms by which NEDD4 expression is associated with BC growth and progression

The mechanisms by which NEDD4 promotes BC growth/progression and contributes to a poor prognosis are not fully understood. NEDD4 functions as an oncogene by facilitating the activation of Akt [13, 21, 22, 38], a protein related to tumor development, drug resistance, and poor prognosis. NEDD4 is reported to increase PI3K–Akt activity during embryonic development via the maintenance of cell surface IGF-1R protein levels. NEDD4 also regulates Akt via PTEN. NEDD4 negatively regulates PTEN by promoting its poly-ubiquitination and subsequent degradation. Increasing levels of NEDD4 significantly reduced PTEN expression, and potentiated cell proliferation and prostate/bladder tumor formation, suggesting an oncogenic role for NEDD4-1 in regulating PTEN functions [13]. Besides degradation, NEDD4 is also involved in the monoubiquitination of PTEN and its ensuing relocalization to the nucleus [43]. However, subsequent studies have shown no difference in the stability and localization of PTEN in two different strains of NEDD4-deficient mice [23]. In addition, NEDD4 is overexpressed in colorectal cancer and promotes colonic cell growth independently of the PI3K/PTEN/AKT pathway [44]. Therefore, regulation of PTEN by NEDD4 may depend on the model system and cellular context used. Our studies are consistent with a previous report describing how NEDD4 expression is not associated with PTEN in human breast carcinoma [15]. Our results suggest that NEDD4 may promote BC growth and progression via an IGF-1R/Akt pathway, even in the cells that harbor an activating PI3K mutation [34]. Although IGF-1R is likely involved in the NEDD4-mediated Akt activation, additional mechanisms cannot be excluded. For instance, Akt activation can be blunted by phosphatases that inhibit Akt activity by dephosphorylation [45]. Therefore, NEDD4 could target phosphatases that directly dephosphorylate Akt, leading to increase of p-Akt, without greatly increased PTEN. The mechanisms by which NEDD4 promotes IGF-1R expression remain unknown. Although NEDD4 regulates IGF-1R in a positive manner by regulating the function of the adaptor protein, Grb10 [17], the latter appears not to be a direct ubiquitination substrate of NEDD4 [46]. Thus, it is most likely that NEDD4 plays an important role in cell proliferation via the activation of IGF-1R signaling by the inhibition of Grb10. NEDD4 may not directly ubiquitinate IGF-1R, but controls the activity of other E3 ligase proteins, which are responsible for Grb10 ubiquitination and degradation. It would be interesting to know if NEDD4 promotes cell proliferation via the facilitation of IGF-1R signaling by the regulation of Grb10 in BC cells.

Despite accumulated findings pointing to tumor-promoting functions, the roles of NEDD4 in cancer appear to be more complex. While NEDD4 functions as an oncogene in most cancers [21, 28, 44, 47, 48], it can also act as a tumor suppressor in some tumors [14, 19, 49]. NEDD4 was recently found to suppress the growth of neuroblastoma and pancreatic cancers by targeting Myc and RAS oncoproteins for ubiquitination and degradation [14, 49]. Therefore, NEDD4 may act as an oncogene in a cellular context-based manner. A recent study also suggested that low NEDD4 expression was closely related to worse outcomes in multiple myeloma [50]. NEDD4 can distinctly regulate degradative ubiquitination of different types of protein substrates in various cancer models, which leads to the promotion or suppression of tumorigenesis. Interestingly, a very recent study suggests that NEDD4 is required for the proteasomal degradation of PIP5Kα, which acts upstream of PI3K/Akt signaling by supplying the PI3K substrate, PIP2, and promoting BC cell proliferation [51]. The nature of the regulation of NEDD4 in targeting PIP5K for degradation in BC growth and progression requires further study.

Lastly, the roles of NEDD4 in the DNA damage response may contribute to the poor prognosis of patients with BC showing a high expression of NEDD4. It has been demonstrated that loss of NEDD4 increased the percentage of G1-arrested cells following a DNA-damaging insult and reduced the cell growth rate, which depends on p53, an important factor of the DNA damage response [52]. NEDD4 contributes to DNA damage–induced cell-cycle arrest and the inhibition of p53-dependent cell growth [52]. Interestingly, Zhou et al. showed that NEDD4 overexpression can sensitize lung cancer cells to apoptosis induced by the DNA-damaging drug, etoposide [53]. Given that DNA damage response and repair are important in any response to genotoxic chemotherapeutic drugs and ionizing radiation, and the resistance/outcomes of treatment by such modalities, it would be of interest to examine how NEDD4 affects signaling induced by DNA damage caused by chemotherapy and ionizing radiation.

The therapeutic potential of targeting the ubiquitin system has been demonstrated by the approval, by the Food and Drug Administration, of the proteasome inhibitor, Velcade, for clinical use. Theoretically, targeting E3 ligases is better than targeting the proteasome because E3 ligases represent the last step of the enzymatic cascade that determines a high degree of specificity and selectivity toward target substrates in cells [54]. Thus, NEDD4 may be a promising target for new cancer therapy.

Several limitations exist in our study. For instance, some subgroups of BC cases were on a relatively small scale and this may have affected our results. The pathological classification of patients with BC using NEDD4 IHC staining scores should be further investigated with an increased number of cases, such as with a more appropriate number of DCIS samples. In addition, TNM stage IV tumors were not included in this study.

## Conclusions

In summary, we demonstrate that NEDD4 promotes BC growth. NEDD4 is markedly overexpressed in BC and is associated with BC progression. Importantly, the upregulation of NEDD4 is associated with a poor prognosis. Investigating the precise role of NEDD4 in BC growth and progression will increase our knowledge of the biological function of NEDD4. Our study has uncovered a new potential target in BC in that targeting E3 ligases offers a promising therapeutic approach for this disease.

## Supplementary information

**Additional file 1: Figure S1.** NEDD4 facilitates proliferation in BC cell lines.

**Additional file 2: Table S1.** Clinicopathological characteristics of patient samples and expression of NEDD4 in BC.

**Additional file 3: Figure S2.** Comparison of NEDD4 mRNA expression in the ER+ and ER- populations of GSE20685.

**Additional file 4: Figure S3.** Correlation of NEDD4 protein and mRNA in TCGA. Utility of NEDD4 expression in the overall, ER-, and ER+ populations of GSE20685.

**Additional file 5: Figure S4.** Prognostic impact of NEDD4 in BC with different ER and Her2 statuses.

**Additional file 6: Figure S5.** Kaplan–Meier analysis of the prognostic utility of NEDD4 expression in the overall, ER-, and ER+ populations of GSE20685.

**Additional file 7: Figure S6.** NEDD4 is not essential for BC migration in in vitro assays.

## Data Availability

All data generated or analyzed during this study are included in this published article and its supplementary information files.

## Abbreviations

ANT: Adjacent normal tissue
BC: Breast cancer
BCLNM: Breast cancer with lymph node metastasis
DCIS: Ductal carcinoma in situ
DFS: Disease-free survival
ER: Estrogen receptor
GAPDH: Glyceraldehyde-3-phosphate dehydrogenase
Her2: Human epidermal growth factor receptor 2
IHC: Immunohistochemistry
IRS: Immunoreactive score
IDC: Invasive ductal carcinoma
IGF-1R: Insulin-like growth factor 1 receptor
NEDD4: Neural precursor cell expressed-developmentally downregulated 4
OS: Overall survival
PBS: Phosphate-buffered saline
PR: Progesterone receptor
PTEN: Phosphatase and tensin homolog
TCGA: The Cancer Genome Atlas
TMA: Tissue microarray
TNM: Tumor–node–metastasis
TNBC: Triple negative breast cancer
