# Supplementary figures and images for: NEDD4 expression is associated with breast cancer progression and is predictive of a poor prognosis
Source: Breast Cancer Res. 2019 Dec 19;21:148. doi: 10.1186/s13058-019-1236-7 (PMC6923956; doi:10.1186/s13058-019-1236-7)

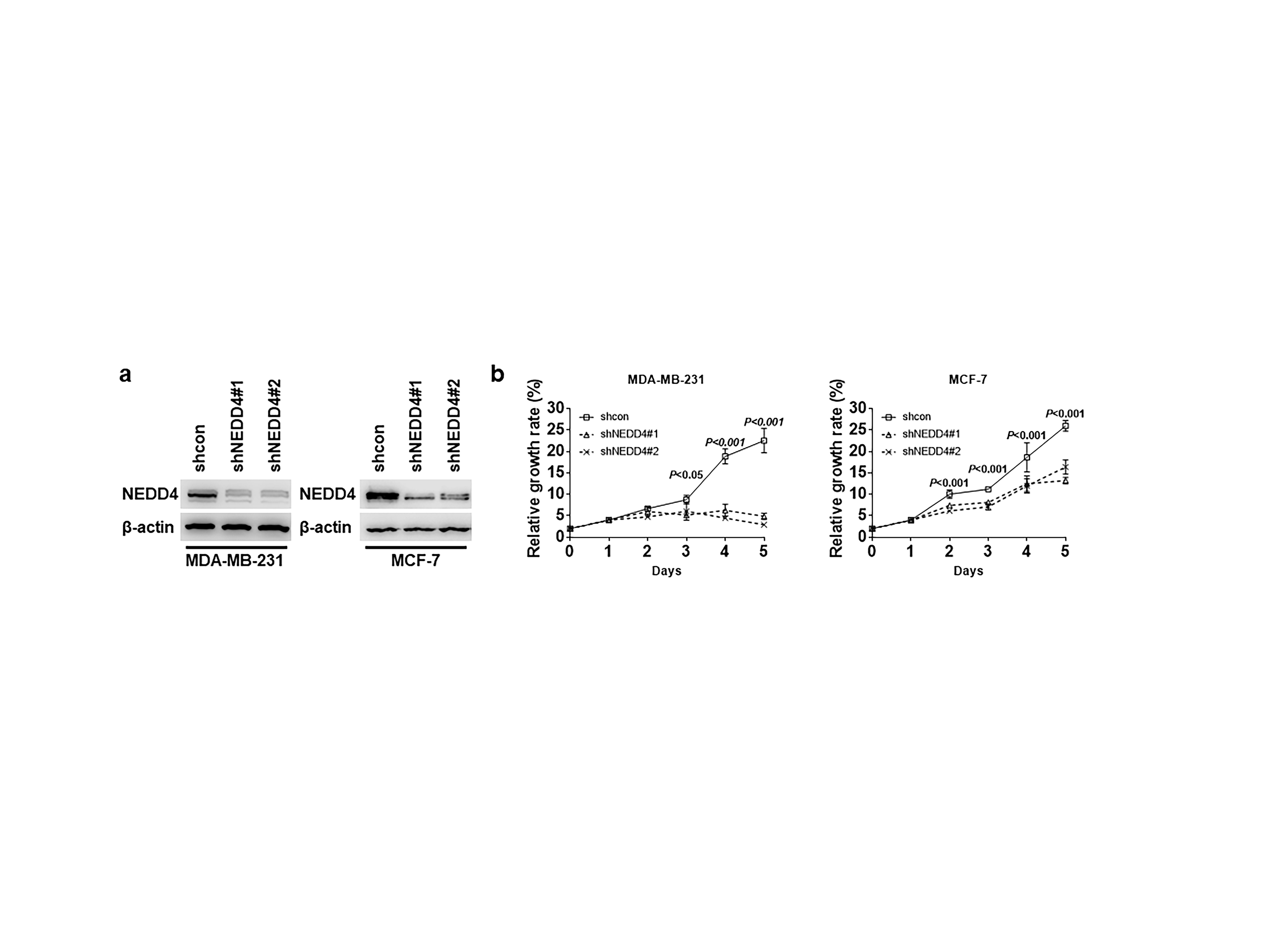

Supplement: Supplementary file 1 — Additional file 1: Figure S1. NEDD4 facilitates proliferation in BC cell lines. [file 13058_2019_1236_MOESM1_ESM.tif]

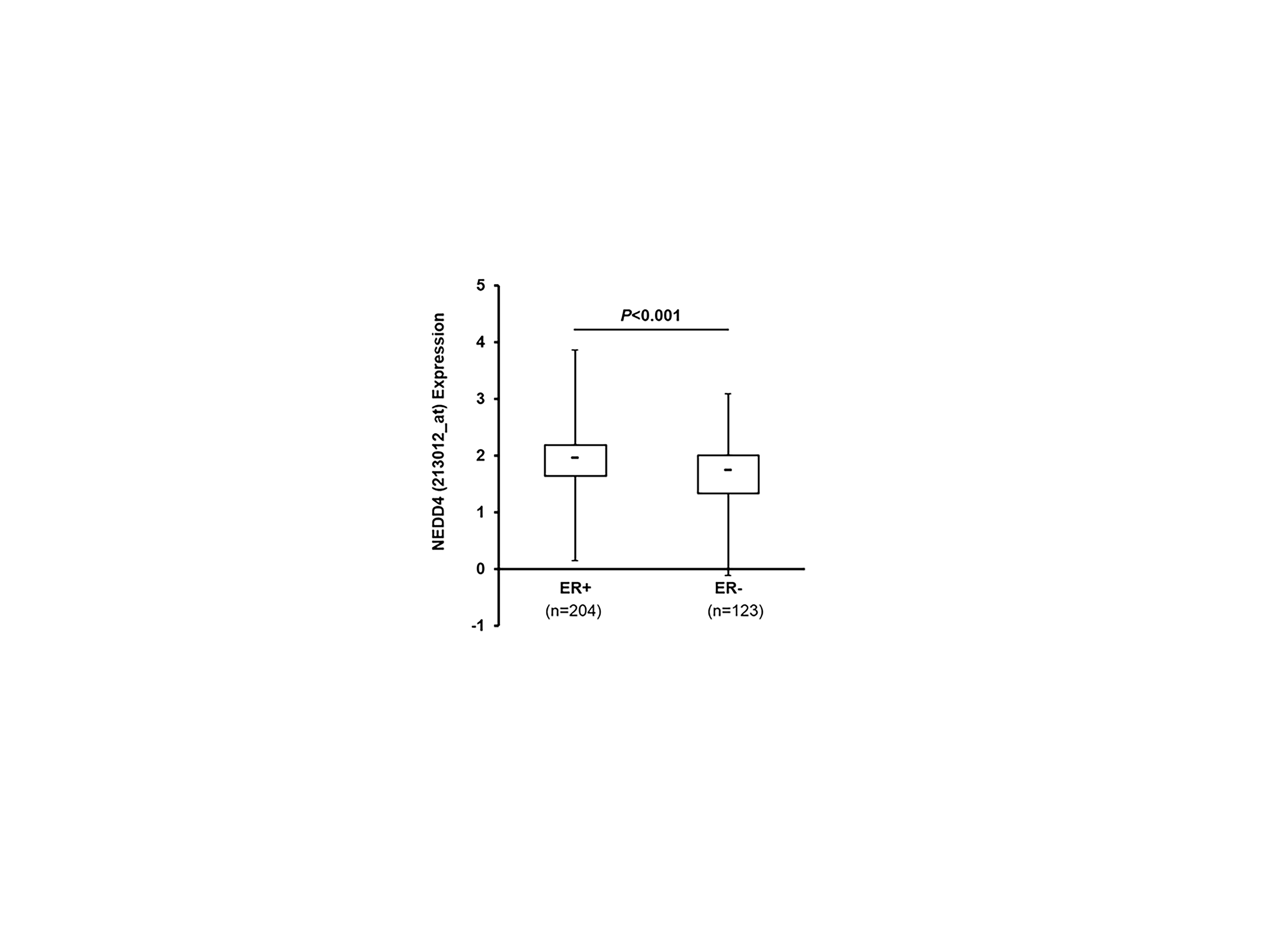

Supplement: Supplementary file 3 — Additional file 3: Figure S2. Comparison of NEDD4 mRNA expression in the ER+ and ER- populations of GSE20685. [file 13058_2019_1236_MOESM3_ESM.tif]

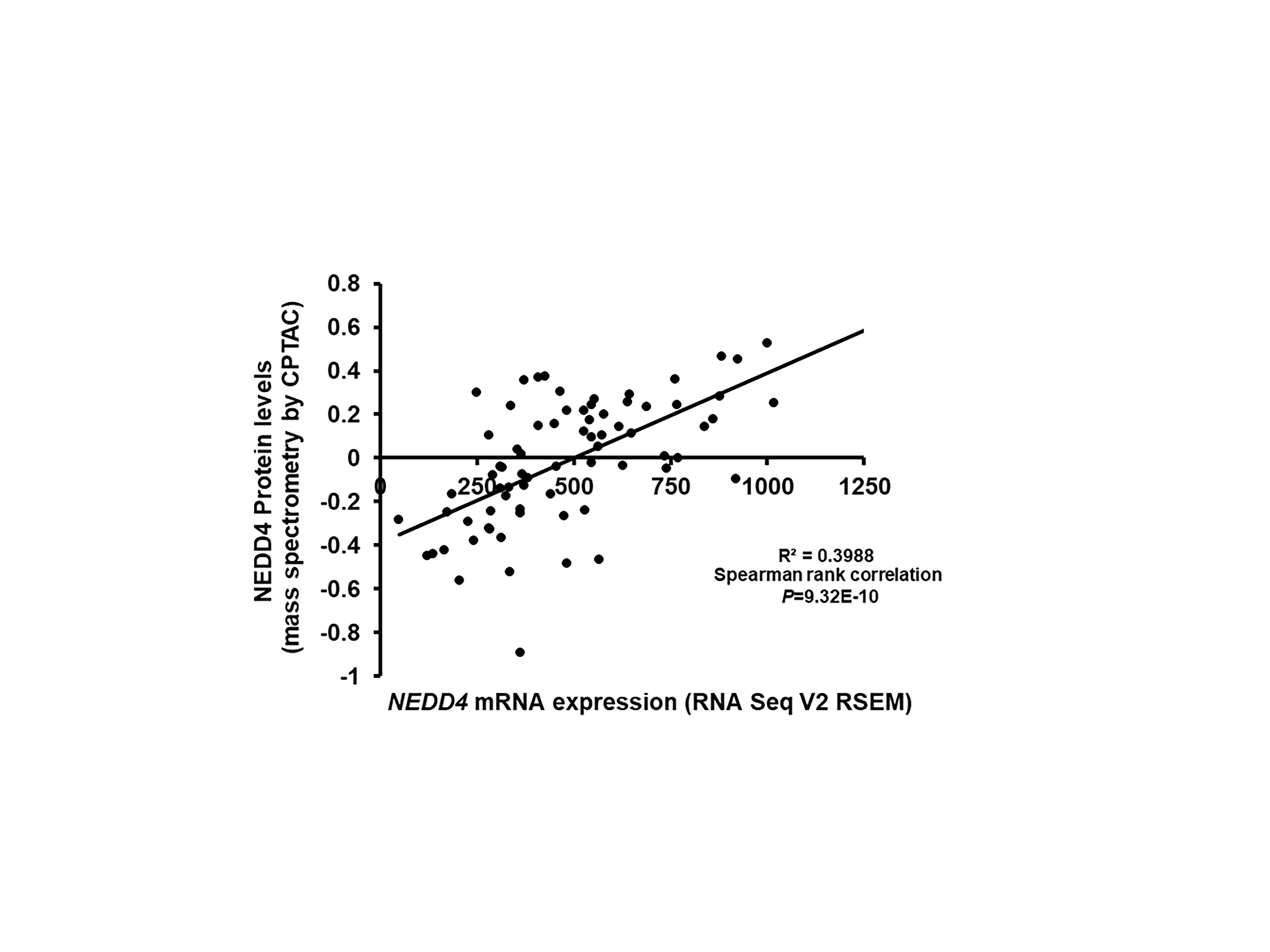

Supplement: Supplementary file 4 — Additional file 4: Figure S3. Correlation of NEDD4 protein and mRNA in TCGA. Utility of NEDD4 expression in the overall, ER-, and ER+ populations of GSE20685. [file 13058_2019_1236_MOESM4_ESM.tif]

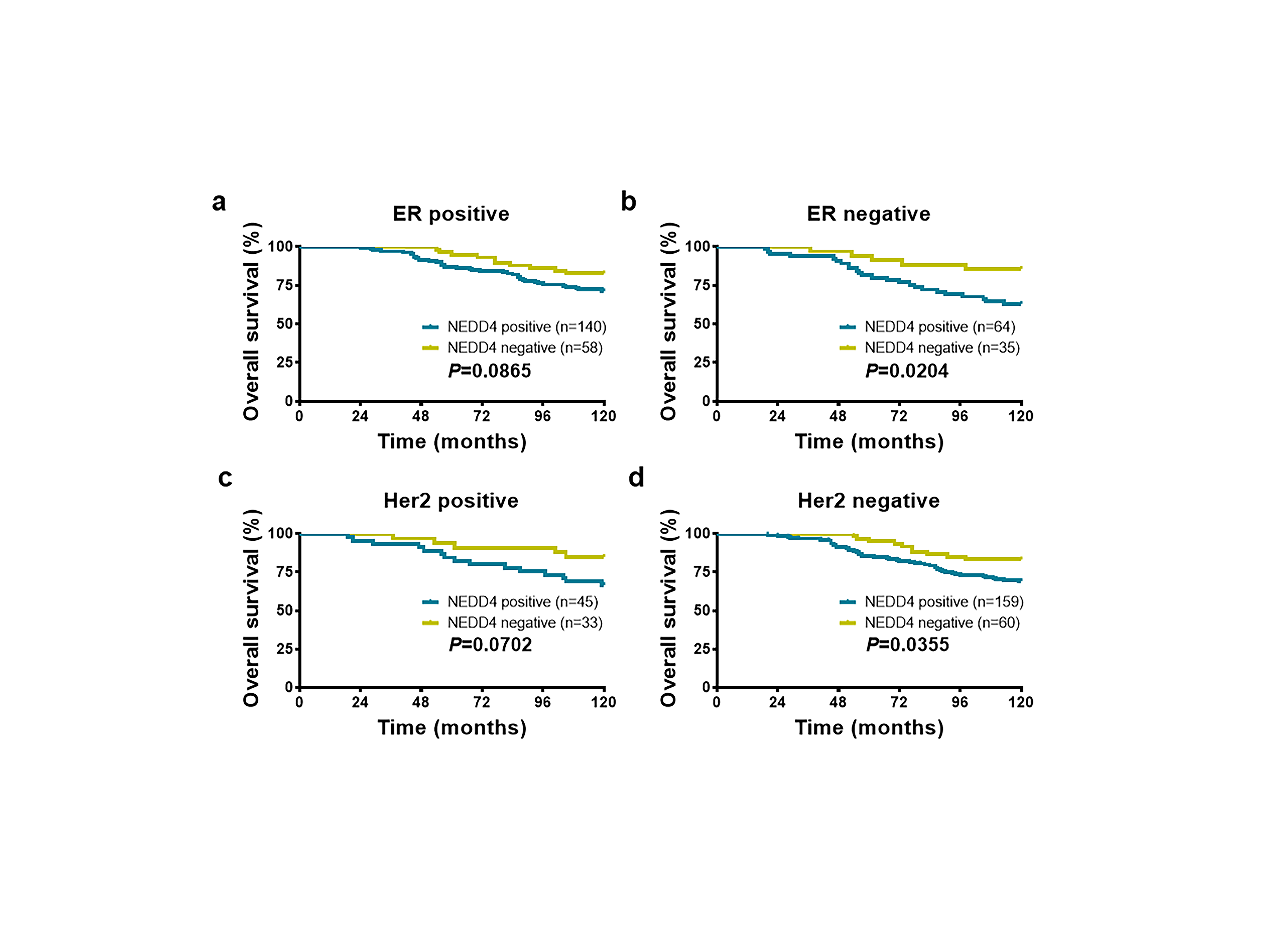

Supplement: Supplementary file 5 — Additional file 5: Figure S4. Prognostic impact of NEDD4 in BC with different ER and Her2 statuses. [file 13058_2019_1236_MOESM5_ESM.tif]

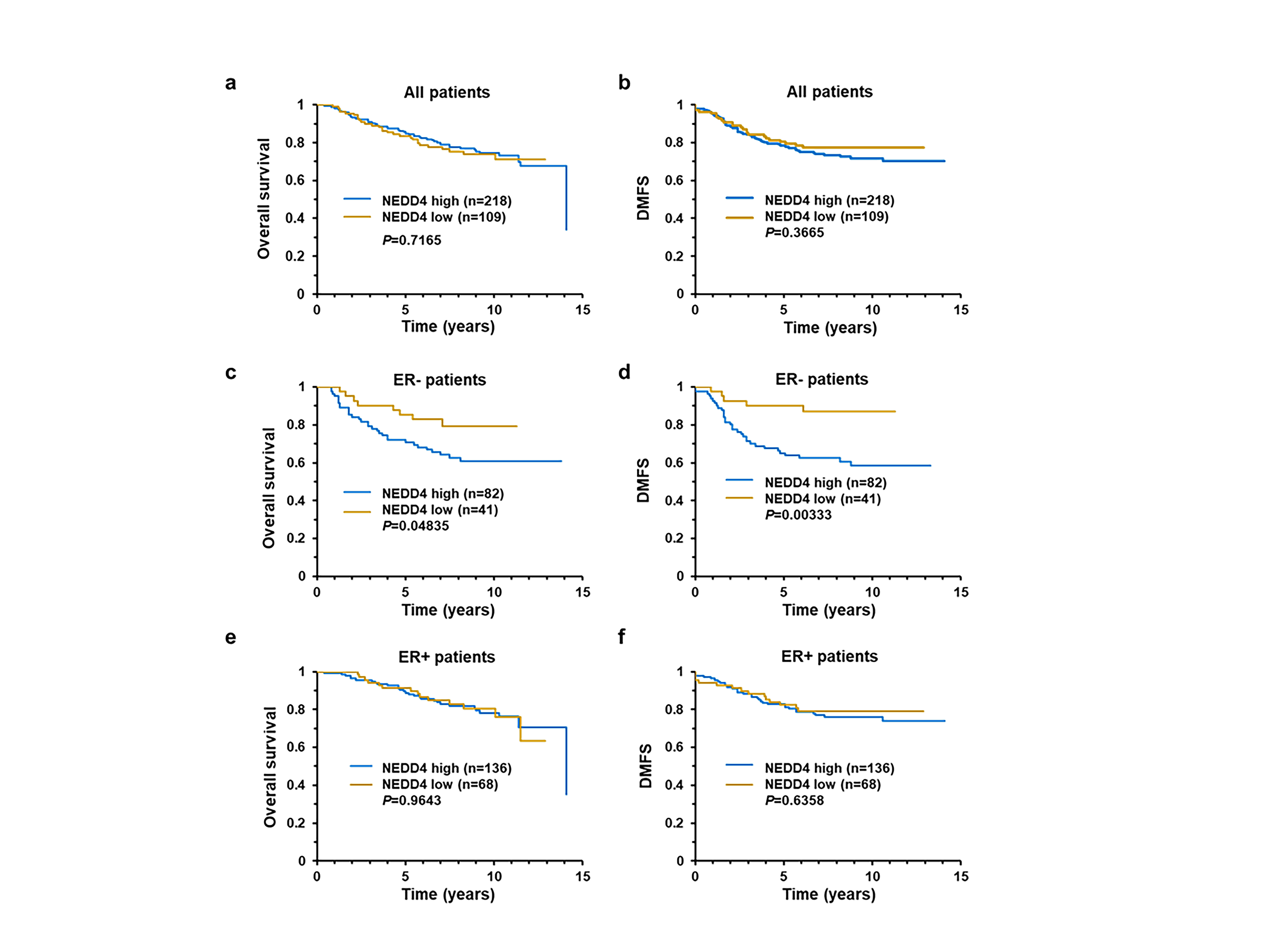

Supplement: Supplementary file 6 — Additional file 6: Figure S5. Kaplan–Meier analysis of the prognostic utility of NEDD4 expression in the overall, ER-, and ER+ populations of GSE20685. [file 13058_2019_1236_MOESM6_ESM.tif]

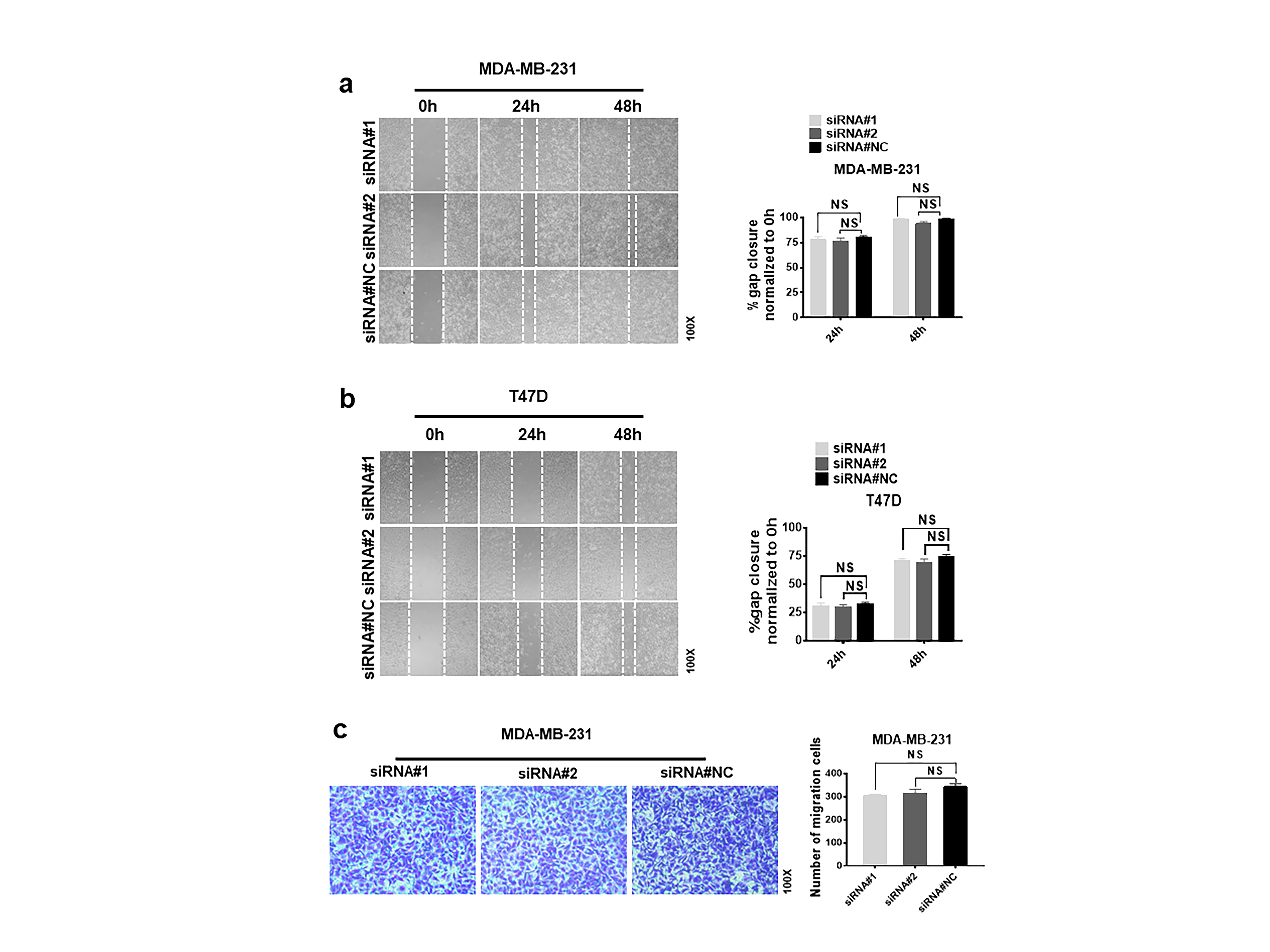

Supplement: Supplementary file 7 — Additional file 7: Figure S6. NEDD4 is not essential for BC migration in in vitro assays. [file 13058_2019_1236_MOESM7_ESM.tif]
