# Supplementary material for: NEDD4 expression is associated with breast cancer progression and is predictive of a poor prognosis
Source: Breast Cancer Res. 2019 Dec 19;21:148. doi: 10.1186/s13058-019-1236-7 (PMC6923956; doi:10.1186/s13058-019-1236-7)
Supplement: Supplementary file 2 — Additional file 2: Table S1. Clinicopathological characteristics of patient samples and expression of NEDD4 in BC. [file 13058_2019_1236_MOESM2_ESM.docx]

**Supplemental Table 1. Clinicopathological characteristics of patient samples and expression of NEDD4**

| **Parameter** | **Number of cases (%)** |
| --- | --- |
| **Gender** |  |
| Male | 0 (0.0) |
| Female | 445 (100.0) |
| **Age (years)** |  |
| ≤35 | 50 (11.0) |
| >35 | 395 (88.0) |
| **Menopausal status** |  |
| Premenopausal | 240 (53.9) |
| Postmenopausal | 205 (46.1 |
| **Tumor size** |  |
| T1a/b | 67(15.1) |
| T1c | 127(28.5) |
| T2 | 220 (49.4) |
| T3 | 31 (7.0) |
| **Histological grade** |  |
| 1 | 39 (8.8) |
| 2 | 204 (45.8) |
| 3 | 144 (32.4) |
| Unknown | 58 (13.0) |
| **Nodal status** |  |
| Negative | 263 (59.1) |
| Positive | 182 (40.9) |
| **TNM stage** |  |
| I | 110 (24.7) |
| II | 255 (57.3) |
| III | 80 (18.0) |
| **ER status** |  |
| Negative | 149 (33.5) |
| Positive | 296 (66.5) |
| **PR status** |  |
| Negative | 139 (31.2) |
| Positive | 306 (68.8) |
| **Her2 status** |  |
| Negative | 325 (73.0) |
| Positive | 120 (27.0) |
| **Ki-67 status** |  |
| ≤20% | 133 (29.9) |
| >20% | 312 (70.1) |
| **P53 status** |  |
| Negative | 104 (23.4) |
| Positive | 299 (67.2) |
| Unknown | 42 (9.4) |
| **Molecular subtype** |  |
| Luminal A | 64 (14.4) |
| Luminal B | 267 (60.0) |
| Her2+ | 73 (16.4) |
| TNBC | 41 (9.2) |
| **Histological type** |  |
| In situ | 37 (8.3) |
| Invasive | 408 (92.7) |
| **Expression of NEDD4** |  |
| Low expression | 161 (36.2) |
| High expression | 284 (63.8) |

Tumor size: 0.1cm<T1a/b<1cm; 1cm≤T1c<2cm; 2cm≤T2<5cm; 5cm≤T3.

Abbreviations:NEDD4, neural precursor cell-expressed developmentally down-regulated gene 4; ER, estrogen receptor; Her2, human epidermal growth factor receptor 2; PR, progesterone receptor; TNBC, triple negative breast cancer; TNM, tumor–node–metastasis.
